# Extracellular volume fraction measurement correlates with lymphocyte abundance in thymic epithelial tumors

**DOI:** 10.1186/s40644-020-00349-4

**Published:** 2020-10-07

**Authors:** Chao-Chun Chang, Chia-Ying Lin, Chang-Yao Chu, Yi-Cheng Hsiung, Ming-Tsung Chuang, Yau-Lin Tseng, Yi-Ting Yen

**Affiliations:** 1 Division of Thoracic Surgery, Department of Surgery, National Cheng Kung University Hospital, College of Medicine, National Cheng Kung University, Tainan, Taiwan; 2 Department of Medical Imaging, National Cheng Kung University Hospital, College of Medicine, National Cheng Kung University, Tainan, Taiwan; 3 Department of Pathology, National Cheng Kung University Hospital, Tainan, Taiwan; 4 Division of Trauma and Acute Care Surgery, Department of Surgery, National Cheng Kung University Hospital, College of Medicine, National Cheng Kung University, No.138, Sheng-Li Road, Tainan, 704 Taiwan

**Keywords:** Extracellular space, Magnetic resonance imaging, Thymic epithelial tumor (TET), World Health Organization (WHO)

## Abstract

**Background:**

Recent advance in tissue characterization with parametric mapping imaging has the potential to be a novel biomarker for histopathologic correlation with thymic epithelial tumors (TETs). The purpose of our study is to evaluate MRI T1 mapping with the calculation of extracellular volume (ECV) fraction for histologic correlation with thymic epithelial tumor based on lymphocyte abundance.

**Methods:**

A retrospective study including 31 consecutive patients (14 men and 17 women, median age, 56 years; interquartile range, 12 years) with TETs was performed. The T1 values and ECV were assessed by using quantitative MRI mapping techniques. Mann-Whitney U test, Kruskal-Wallis H test, and receiver operating characteristic curve analyses were used to assess discrimination between different types of TETs based on lymphocyte abundance.

**Results:**

Extracellular volume was significantly higher in TETs with sparse lymphocyte, including type A, type B3, and thymic carcinoma, compared with those with abundant lymphocyte, including type B1, B2, and AB thymomas (42.5% vs 26.9%, respectively; *p* < 0.001). Extracellular volume was significantly higher in thymic carcinoma compared with low grade and high grade thymomas (48.6% vs 31.1% vs 27.6%, respectively; *p* = 0.002).

**Conclusions:**

T1 mapping with the calculation of extracellular volume (ECV) fraction correlate with the WHO histologic classification of thymic epithelial tumor based on lymphocyte abundance.

## Introduction

Thymic epithelial tumors (TETs), including thymoma and thymic carcinoma, show a broad spectrum of histologic features and oncologic behavior. Several classifications have been proposed to correlate the histopathology and the clinical course of the TETs and to reflect their invasiveness and prognosis. The World Health Organization (WHO) histologic consensus classification, proposed in 1999 and revised in 2004, is the currently advocated classification. It represents both the clinical and the functional characters of TETs and hence contributes to the clinical assessment and treatment of the patients [1, 2].

In the WHO classification, the thymomas can be divided into those with spindled neoplastic epithelial cells (A, AB) and those with epithelioid neoplastic epithelial cells (B1-B3). The further subdivision depends on the neoplastic epithelial cells and nonneoplastic immature T-cells component; in type A and B3 thymoma, there is a paucity or even lack of immature T-cells throughout the densely packed spindle cells (type A) or sheets of polygonal tumor cells (type B3), whereas there is abundance of immature T-cells and tumor cells in type AB, B1, and B2 thymomas. Type AB, B1, and B2 thymomas show an abundance of immature lymphocytes (“thymocytes”) either diffusely (all type B1 and B2, rare type AB thymomas) or focally (most type AB thymoma) [3]. Accordingly, TETs were divided into lymphocyte abundant and lymphocyte sparse subgroups based on histopathological findings. The lymphocyte sparse group contains type A, B3 thymomas and thymic carcinoma, whereas the lymphocyte abundant group composes of type B1, B2, and AB thymomas. The clinical outcomes of TETs have been reported to associate with the WHO classification, where type A, AB, and B1 thymomas have better prognosis than type B2, B3 thymomas and thymic carcinoma [2].

Although the WHO classification of TETs has been reported to correlate with clinical outcomes, the histological typing of TETs still remains a challenge for surgical pathologists. Therefore the need for clinical judgement based on a complete history and physical examination, correlated with laboratory tests and radiological features, helps to develop a presumptive diagnosis. TETs can be divided into two compartments: intracellular cellular volume (ICV) and extracellular cellular volume (ECV). While the value of ICV represents tumor cell and lymphocyte, the value of ECV is composed of the measurement of extracellular matrix and intracapillary plasma volume. T1 mapping with ECV fraction measurement is a feasible and noninvasive clinical tool to assess and quantify tissue composition [4]. Compared with the sole evaluation of T1 mapping, ECV shows advantage including independence of field strength, imaging parameters and contrast dose because it is a ratio derived from pre- and post- contrast T1 values in addition to the physiologically intuitive unit of measurement [5, 6]. To the best of our knowledge, there has been no study applying T1 mapping with ECV fraction measurement to the diagnosis of TETs. The aim of this study is to assess the diagnostic feasibility of T1-mapping with ECV fraction measurement for the evaluation of TETs. We hypothesize that ECV correlates with the WHO classification because it reflects stroma-cell ratio.

## Material and methods

### Study population

This retrospective study was approved by the institutional review board (B-ER-108-046) and informed consent was waived. Between January 2018 and October 2019, a total of 31 consecutive patients with TET more than 2 cm in diameter were referred for mediastinal MR. All the patients underwent thymectomy and thymothymectomy (Video-assisted thoracic surgery, *n* = 17; sternotomy, *n* = 8) or core needle biopsy (*n* = 6), without neo-adjuvant treatments. Data were collected on age, sex, myasthenia gravis symptoms, tumor size, WHO histology classification, Masaoka-Koga stage, history of extrathymic malignancies, and cancer treatments before and after surgery for TETs. The hematocrit (Hct) was measured to calculate the ECV of the tumor.

### Mediastinal MRI acquisition

Mediastinal MR was performed using a 3 Tesla system (Ingenia, Philips Healthcare, Best, the Netherlands) using a 16-channel dStream anterior coil and a 12-channel dStream posterior coil for signal reception. All patients underwent a clinical routine mediastinal image protocol and in addition received native and postcontrast modified Look-Locker inversion-recovery (MOLLI) sequence of the mediastinal tumor. The routine image protocol included axial pre-contrast modified Dixon (mDixon, water, inphase, outphase images), sagittal fat-suppressed T2-weighted, axial ECG-gated breathhold T2 turbo spin echo images with double inversion recovery, axial diffusion-weighted imaging (DWI) (b-values = 0, 400, and 800 s/mm^2^), from which apparent diffusion coefficient (ADC) map was constructed, axial and sagittal T1-weighted imaging after administration of contrast medium. The detail of the protocol was shown in Supplementary Table 1.

A breath-hold, electrographic-gated, modified Look-Locker inversion-recovery (MOLLI) sequence with a 5 s (3 s) 3 s and 4 s (1 s) 3 s (1 s) 2 s sampling pattern was performed for native and post-contrast T1 mapping in the axial orientation, respectively, with a balanced steady-state free-precession (bSSFP) readout, FOV 250 × 250 mm^2^, matrix 192 × 192, TR/TE 2.8/1.29 ms, acquisition window duration 165 ms, flip angle 35 degrees and 7 mm thickness.

T1 maps were acquired before and after 10 min after a bolus contrast agent administration (0.1 mmol/kg; Gadovist, Bayer Healthcare, Leverkusen, Germany). T1 maps were generated online from the MOLLI images after the motion correction.

### Image analysis

**Fig. 1:**
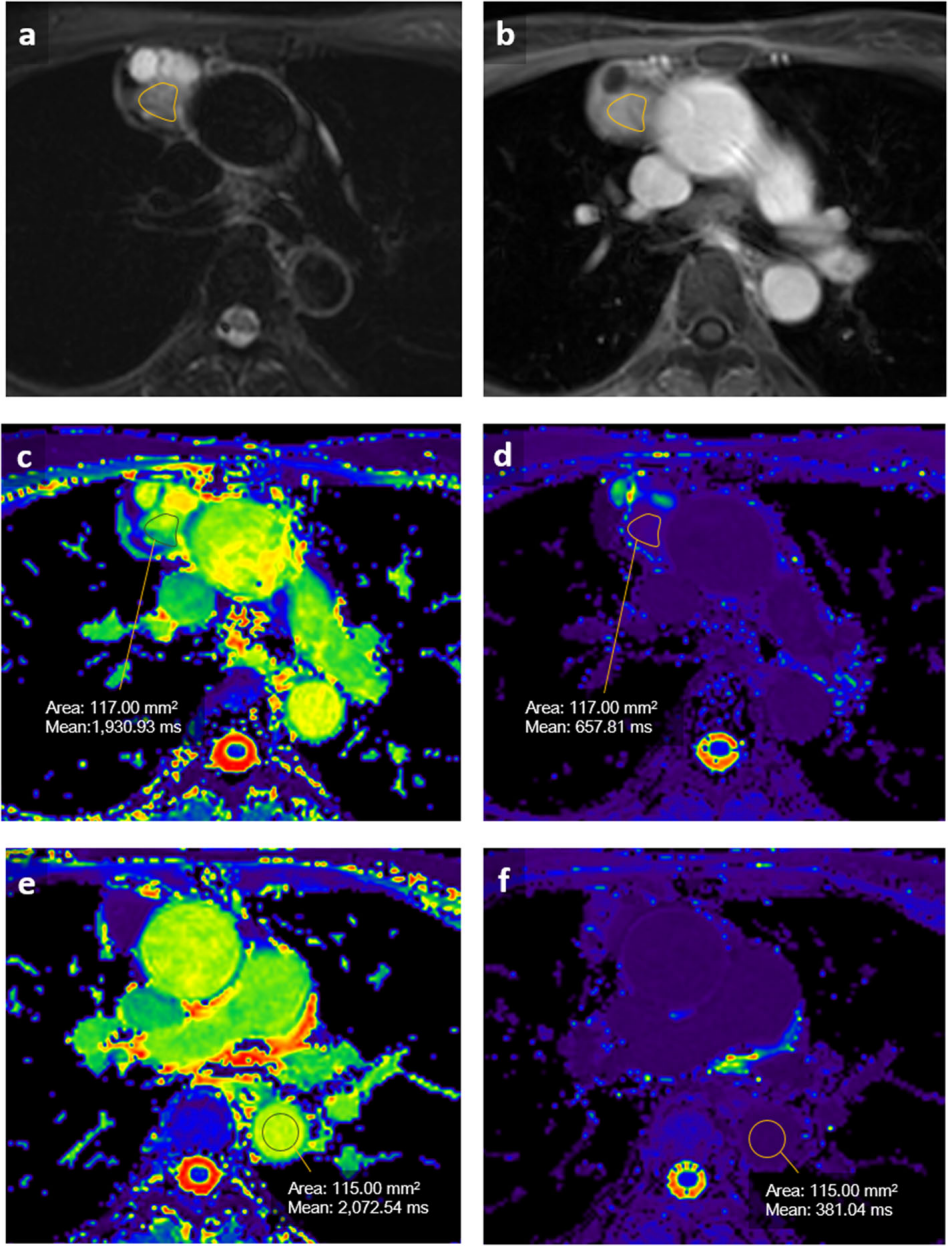
Representative example of a 59-year-old female with type A thyoma. **a** Axial T2-weighted black-blood imaging. **b** Axial post-gadolinium T1-weighted images (T1WI). **c, e** Axial native T1 mapping imaging. **d, f** Axial post-contrast T1 mapping imaging. The freehand region of interest (ROIs) were manually drawn on both native T1 and post-contrast T1 mapping imaging (**c, d**) where the largest area of the tumor on axial MR images was included. The ROI was smaller in size than the mass and included only the enhancing part of the tumor avoiding cystic or necrosis part. T1 values of the blood pool were obtained from the descending thoracic aorta at right pulmonary artery level on both native T1 and post-contrast T1 mapping imaging (**e, f**)

All examinations were independently analyzed by a board-certified radiologist (C.Y.L., with 5 years of experience in thoracic MRI), blinded to patient’s information and clinical data. The longest tumor diameter was measured at the widest dimension on transverse cross-sectional images. Tumor boundaries were determined and segmented via inspection of T2-weighted, contrast enhanced T1-weighted, and DW imaging. For each patient, a freehand region of interest (ROIs) were manually drawn on the postcontrast sequences in conjunction with T2-weighted imaging on three consecutive levels where the largest area of the tumor on axial MR images was included. In order to avoid including cystic or calcified part, necrosis, or hemorrhage of the tumor, the ROI was manually placed because of current development limitation of technology. The ROI was smaller in size than the mass and included only the enhancing part of the tumor. The respective ROI was then copied to the MOLLI sequence and ADC maps, using an automatic coregistration tool and by visual correlation in case of breathing artifacts. For each ROI, mean T1 value was recorded and used for final analysis. T1 values of the blood pool were obtained from the descending thoracic aorta at right pulmonary artery level on the transversal maps (Fig. 1). Extracellular volume values were normalized for hematocrit and calculated from pre- and postcontrast T1 values by using the following equation:

$$ \mathrm{ECV}=\left(1-\mathrm{hematocrit}\right)\ \frac{\frac{1}{post\ contrast\ T1\  tumor}-\frac{1}{native\ T1\  tumor}}{\frac{1}{post\ contrast\ T1\  blood}-\frac{1}{native\ T1\  blood}} $$

The calculation of ECV assumes an equilibrium of gadolinium-based contrast agents between the ECV and intravascular compartment.

### Interobserver concordance

For native T1 value and ECV fraction measurement, another rater (C.C.C.), a board-certified thoracic surgeon with 5 years of experience in thoracic MRI, who was blinded to the patient’s clinical information, also delineated the ROIs. The measurements from the second rater were only used to compare with the findings of the radiologist to assess the inter-observer concordance.

### Pathologic diagnosis

The final diagnosis was confirmed on microscopic pathologic examination. The specimens of TETs were fixed in 10% formalin and stained with conventional hematoxylin-eosin staining. Pathologic analysis was performed by a board-certified pulmonary pathologist (C.Y.C., with 7 years of experience), who was blinded to the clinical and MR findings. Thymic epithelial tumors were classified on the basis of the morphologic analysis of the neoplastic epithelial cells with lymphocyte-epithelial cell ratio based on the WHO histologic classification. Further division into lymphocyte sparse group (including type A, B3 thymomas and thymic carcinoma) and lymphocyte abundant group (including type B1, B2, and AB thymomas) was performed based on histopathological findings. Thymic epithelial tumors were staged according to the Masaoka-Koga clinical staging system.

### Statistical analysis

Descriptive statistics were displayed as the median with 1st and 3rd quartile values. The Mann-Whitney U test and Kruskal-Wallis H test were used to compare variables. The nonparametric receiver operating characteristic analysis was performed to assess the discriminative ability, and area under the receiver operating characteristic curve (AUC) was calculated. Optimal cutoff values were derived from receiver operating characteristic curves, and sensitivity and specificity were calculated based on these best cutoff values. Inter-observer reliability was assessed with the interclass correlation coefficient (ICC) based on a two-way random-effects model of *absolute agreement*. A *p* value of ≤0.05 was set to indicate statistical significance. SPSS system (IBM SPSS Statistics, Version 22.0, Armonk, NY) was used for statistical analysis.

## Results

### Demographic data

Demographic characteristics are summarized in Table 1 and detailed in Supplementary Table 2. A total of 31 patients were included in this study, 14 men (45.2%) and 17 women (54.8%). Only one patient had extrathymic malignancy as endometrial cancer. Four patients (12.9%) had myasthenia gravis symptoms. The median age was 56.0 years old. The median size measured on the largest tumor-containing axial slice was 5.75 cm. The Masaoka-Koga stage of the patients showed 11 (35.5%) stage I, 7 (22.6%) stage II, 5 (16.1%) stage III, 8 (25.8%) stage IV. 18 (58.1%) patients had noninvasive TETs, while 13 patients (41.9%) had invasive TETs. Six patients received chemotherapy without surgery because of great vessel invasion. In patients undergoing surgery, 8 had sternotomy, and 17 had video-assisted thoracoscopic surgery (VATS). The number of type A, AB, B1, B2, B3, and thymic carcinoma were 4 (12.9%), 7 (22.6%), 2 (6.5%), 9 (29.0%), 1 (3.2%), and 8 (25.8%), respectively. Thirteen patients (41.9%) had low grade thymoma, 10 patients (32.3%) had high grade thymoma, and 8 (25.8%) patients had thymic carcinoma according to the histologic grading. There was no atypical type A thymoma, micronodular thymoma, or metaplastic thymoma. All thymic carcinoma patients were squamous cell carcinoma. Based on lymphocyte abundance, 13 patients (41.9%) had lymphocyte sparse TETs and 18 patients (58.1%) had lymphocyte abundant TETs. Table 2 shows the relationship between the WHO classification and clinical staging of TETs.

**Table 1:** Demographic data

Variables	Number (*n* = 31)
**Gender, male (%)**	14 (45.2%)
**Age**^a^	56.00 (52.00, 64.00)
**MG**	4 (12.9%)
**Tumor size (cm)**^a^	5.75 (4.50, 8.88)
**First-line treatment**	
VATS	17 (54.8%)
Sternotomy	8 (25.8%)
Chemotherapy	6 (19.4%)
**Mosaoka-Koga stage**	
I	11 (35.5%)
II	7 (22.6%)
III	5 (16.1%)
IV	8 (25.8%)
**WHO classification**	
A	4 (12.9%)
AB	7 (22.6%)
B1	2 (6.5%)
B2	9 (29.0%)
B3	1 (3.2%)
Carcinoma	8 (25.8%)

Note.—Unless otherwise indicated, data are the number of patients, with the percentage in parentheses. VATS = video-assisted thoracic surgery

^a^ Data are the median, with the first and third quartile in parentheses

**Table 2:** Relationship between the WHO Classification and Clinical Staging

		Mosaoka-Koga stage			
		I	II	III	IV
**WHO classification**	A	3	1	0	0
	AB	5	2	0	0
	B1	0	1	0	1
	B2	2	3	1	3
	B3	1	0	0	0
	Carcinoma	0	0	4	4

### Native T1 value of Thymic epithelial tumors

The noninvasive TETs showed higher native T1 value than invasive TETs [1574.5 (1338.4, 1694.8) vs. 1384.1 (1309.1, 1458.0), *p* = 0.01]. No significant difference was found in native T1 value between different tumor sizes, presence of myasthenia gravis symptoms, histologic grade and lymphocyte abundance of TETs.

### ECV fraction measurement of Thymic epithelial tumors

**Fig. 2:**
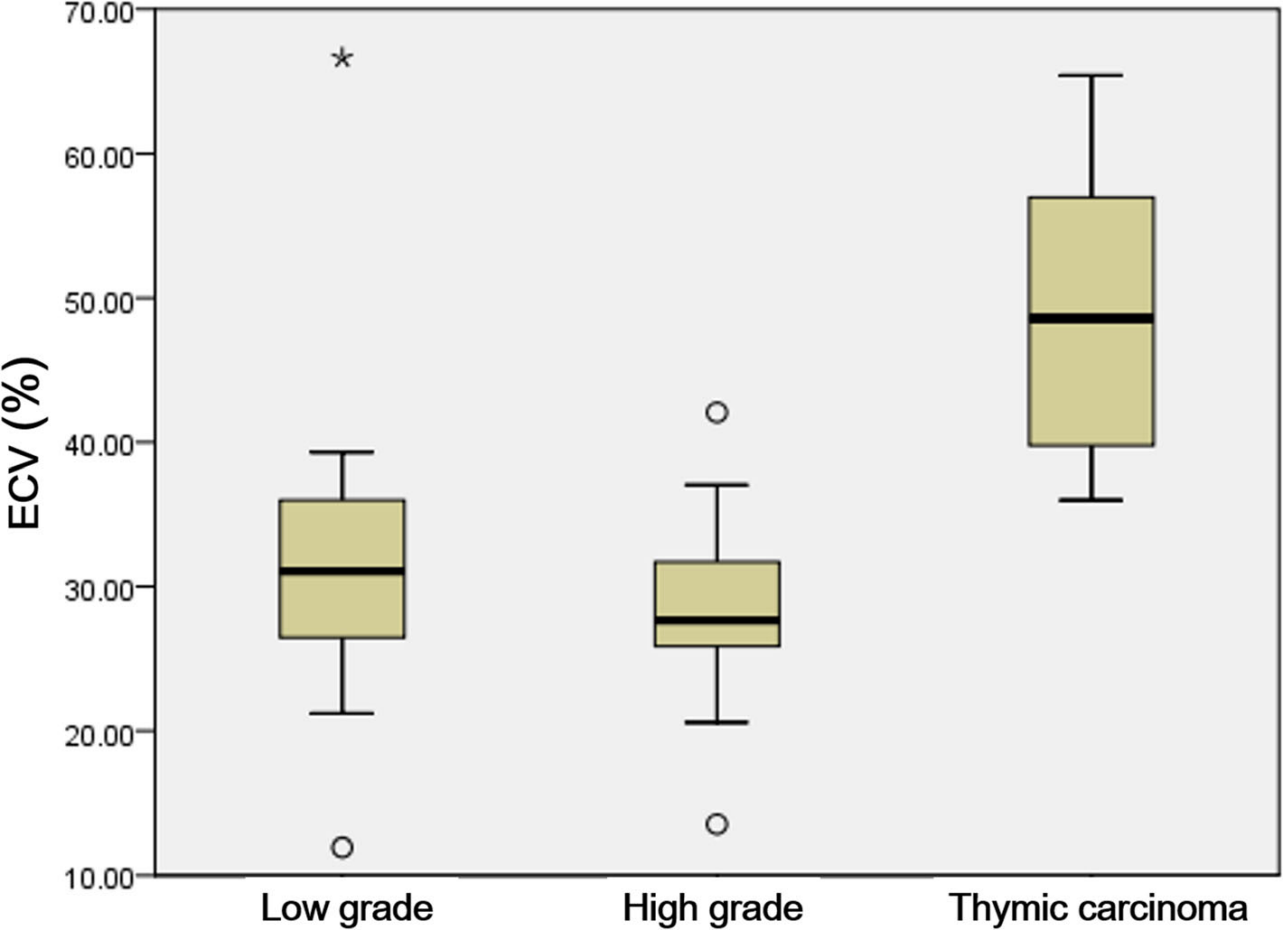
Box and whisker plots show comparison of ECV fractions of low grade TETs, high grade TETs and thymic carcinoma. Higher ECV value was found in thymic carcinoma than high grade and low grade thymomas (*p* = 0.002)

**Fig. 3:**
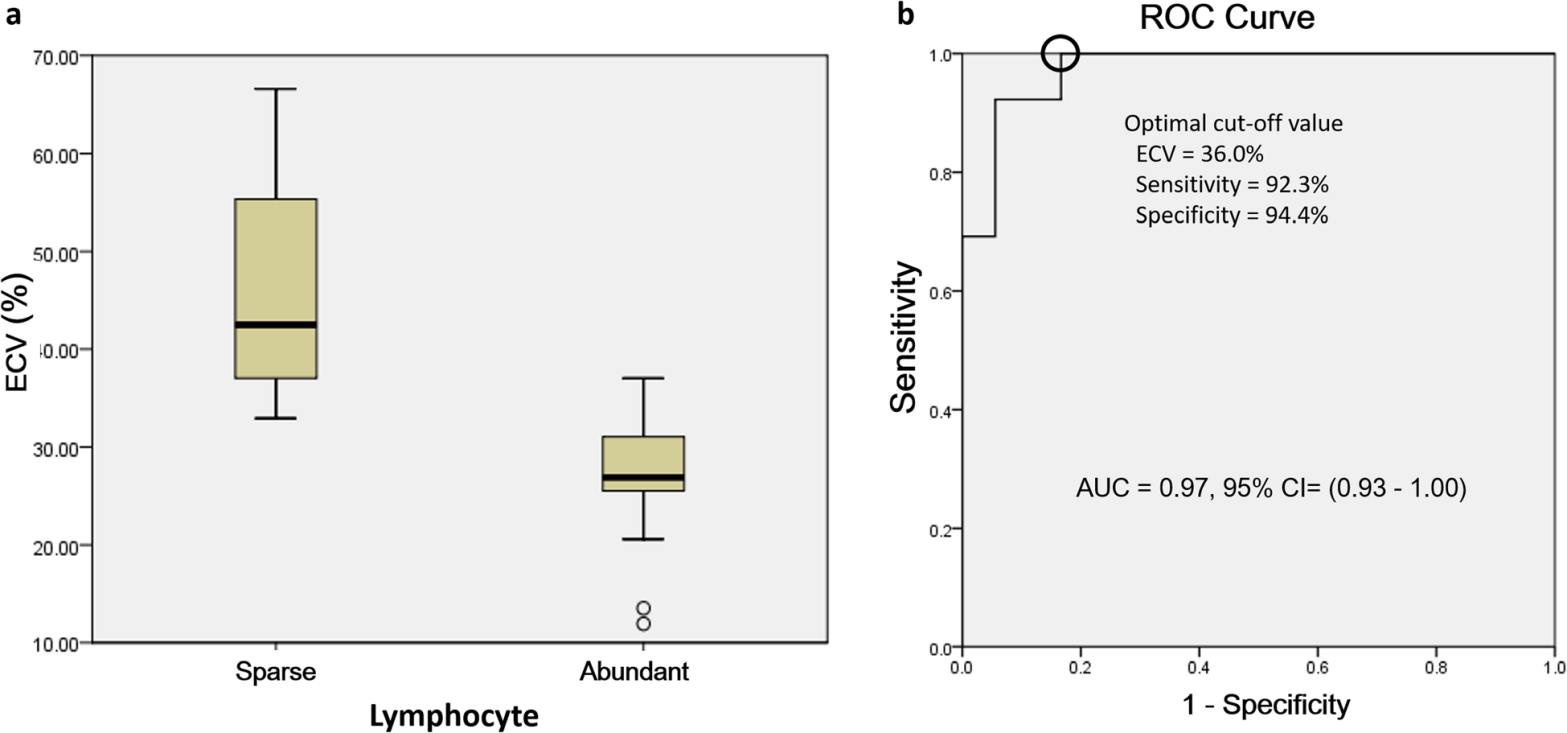
**a** Box and whisker plots show comparison of ECV fractions of TETs based on lymphocyte abundance. Higher ECV was found in lymphocyte sparse TETs than in lymphocyte abundant TETs (*p* < .001). **b** Receiver operating characteristic (ROC) curve shows the cutoff ECV value used to differentiation between the lymphocyte sparse and lymphocyte abundant groups was 36.0%, with sensitivity 92.3%, specificity 94.4%, and area under the curve of 0.97

No significant difference was found in ECV fraction values between different tumor sizes, presence of myasthenia gravis symptoms or tumor invasiveness. The ECV range of TETs were as follows: type A thymoma = 32.93–66.62; type AB = 25.51–36.00; type B1 = 11.93–21.22; type B2 = 13.53–37.05; type B3 = 42.06; thymic carcinoma = 36.00–65.41. The comparison of mean ECV fraction values of low grade TETs, high grade TETs and thymic carcinoma was demonstrated in Fig. 2. Higher ECV value was found in thymic carcinoma than high grade and low grade thymomas [48.6 (38.4, 57.8) vs. 27.6 (24.5, 33.0) vs. 31.1 (26.0, 36.4), *p* = 0.002], and Dunn’s post hoc tests showed significant difference between thymic carcinoma and high grade thymoma (*p* = 0.002) and between thymic carcinoma and low grade thymoma (*p* = 0.011). There wasn’t significant difference between low grade thymoma and high grade thymoma (*p* = 1.000). Higher ECV value was identified in lymphocyte sparse than lymphocyte abundant TETs [42.5 (36.9, 57.0) vs. 26.9 (24.4, 31.2), *p <* 0.001] as shown in Table 3. The comparison of mean ECV fraction values of lymphocyte sparse with lymphocyte abundant TETs was demonstrated in Fig. 3a. Its best cutoff value for differentiation between lymphocyte sparse and abundant tumor was 36.0% at receiver operating characteristic curve analysis, with a sensitivity of 92.3% and a specificity of 94.4% (area under curve 0.97; 95% confidence interval: 0.93–1.00) as shown in Fig. 3b.

**Table 3:** Native T1 value with ECV fraction measurement and ADC value of Thymic Epithelial Tumors

Variables	Native T1 (ms)	***p***	ECV (%)	***p***	ADC (× 10^**−3**^ mm^**2**^/s)	***p***
**Tumor size**		.224		.656		.008
Size ≦ 5 cm (*n* = 12)	1543.9 (1387.51663.3)		31.4 (26.0,38.7)		1.98 (1.51,2.47)	
Size > 5 cm (*n* = 19)	1445.6 (1290.51570.2)		36.0 (26.2,42.5)		1.19 (0.97,1.90)	
**Myasthenia gravis**		.814		.316		.195
No (*n* = 27)	1470.4 (1339.0,1662.5)		33.8 (26.5,42.5)		1.36 (1.08,2.13)	
Yes (*n* = 4)	1461.1 (1248.91645.9)		27.6 (25.7,35.0)		2.02 (1.54,2.42)	
**Invasiveness**		.010		.150		.004
Noninvasive (*n* = 18)	1574.5 (1388.41694.8)		31.4 (26.1,36.8)		2.00 (1.33,2.55)	
Invasive (*n* = 13)	1384.1 (1309.11458.0)		37.0 (27.6,52.2)		1.16 (0.96,1.45)	
**Histologic grade**		.139		.002		.391
Low grade (*n* = 13)	1570.2 (1309.81710.4)		31.1 (26.0,36.4)		1.90 (1.20,2.59)	
High grade (*n* = 10)	1522.8 (1420.71602.6)		27.6 (24.5,33.0)		1.44 (1.06,2.39)	
Carcinoma (n = 8)	1361.6 (1299.81440.5)		48.6 (38.4,57.8)		1.28 (1.02,1.69)	
**Lymphocyte**		.575		<.001		.810
Sparse (n = 13)	1436.3 (1309.11695.7)		42.5 (36.9,57.0)		1.44 (1.19,2.00)	
Rich (n = 18)	1501.9 (1356.91662.7)		26.9 (24.4,31.2)		1.58 (1.06,2.50)	

Note.—Data are the median, with the first and third quartile in parentheses

### ADC value

The mean ADC value was significant lower in tumor sized greater than 5 cm compared with tumor sized less than 5 cm [1.19 (0.97, 1.90) versus 1.98 (1.51, 2.47), *p* = 0.008]. The mean ADC value was significant lower in invasive group compared with noninvasive group [1.16 (0.96, 1.45) versus 2.00 (1.33, 2.55), *p* = 0.004]. There was no significant difference in clinical myasthenia gravis symptoms, histologic grade, or lymphocyte abundance of the tumors. (Table 3).

### Reproducibility assessment

Excellent inter-observer reproducibility was seen in the measurement of the native T1 value and ECV (ICCs = 0.912 and 0.901, respectively).

## Discussion

We have demonstrated in this study that higher ECV value was identified in thymic carcinoma than thymomas, and in lymphocyte sparse TETs than in lymphocyte abundant TETs. A few prior studies analyzed the imaging characteristics of TETs [7–10]. Jeong et al. found that CT imaging findings including irregular contours and necrotic part were more often seen in thymic carcinoma, whereas complete capsule, septum, and homogenous enhancement were more often seen in low grade thymoma [7]. Magnetic resonance (MR) images have been considered better in depicting tumor capsule, septum, or hemorrhage than images of computed tomography (CT) [9]. Recently, quantitative MRI has been increasingly applied to characterize anterior mediastinal tumors [9, 11–17]. Abdel Razek A.A. et al. [12] found that lower ADC value was identified more in high risk thymoma and thymic carcinoma than low risk thymoma. The results can be explained by that decreased diffusion space of water protons in the extracellular and intracellular dimensions due to enlarged nuclei, hyperchromatism, and hypercellularity in high risk thymoma and thymic carcinoma. They also found that ADC value was significantly lower in invasive thymoma than noninvasive thymoma. According to our result, ADC value could only differentiate the invasiveness of TETs but not the histologic types. The variation of the aforementioned results could be attributed to the difference of patient cohorts in each study. In Abdel Razek A.A. et al. study, most high grade thymoma were invasive thymoma (7 invasive thymomas out of 9 high grade thymomas). On the contrary, in our study, we had only 4 invasive thymomas out of 10 high grade thymomas, while the other six were noninvasive thymomas. Our results suggested that ADC value had better correlation with Masaoka stage than histologic type.

Compared with ADC value quantification, T1 mapping imaging with ECV fraction calculation shows the advantage of better spatial resolution, repeatability, reproducibility, and accuracy. The ADC map of TETs shows prominent imaging distortion and prone to motion artifacts, and the small sized tumor cannot be evaluated precisely [18, 19]. On the other hands, there is less image distortion and motion artifact in the breath-hold, EKG-gated T1 mapping with ECV fraction calculation. Even small sized tumor can be adequately measured. In addition, ADC value is affected by different field strength and diffusion encoding technique [20]. On the contrary, ECV calculations represent the ratio of T1 values, and are less sensitive to systemic biases that are likely to cancel one another in the mathematical derivation of ECV. Therefore, we consider ECV fraction a better non-invasive predictive imaging tool than ADC value for histopathological correlation. The addition of T1 mapping and ECV into the routine MR imaging of thymic epithelial tumors may improve assessment of these lesions.

**Fig. 4:**
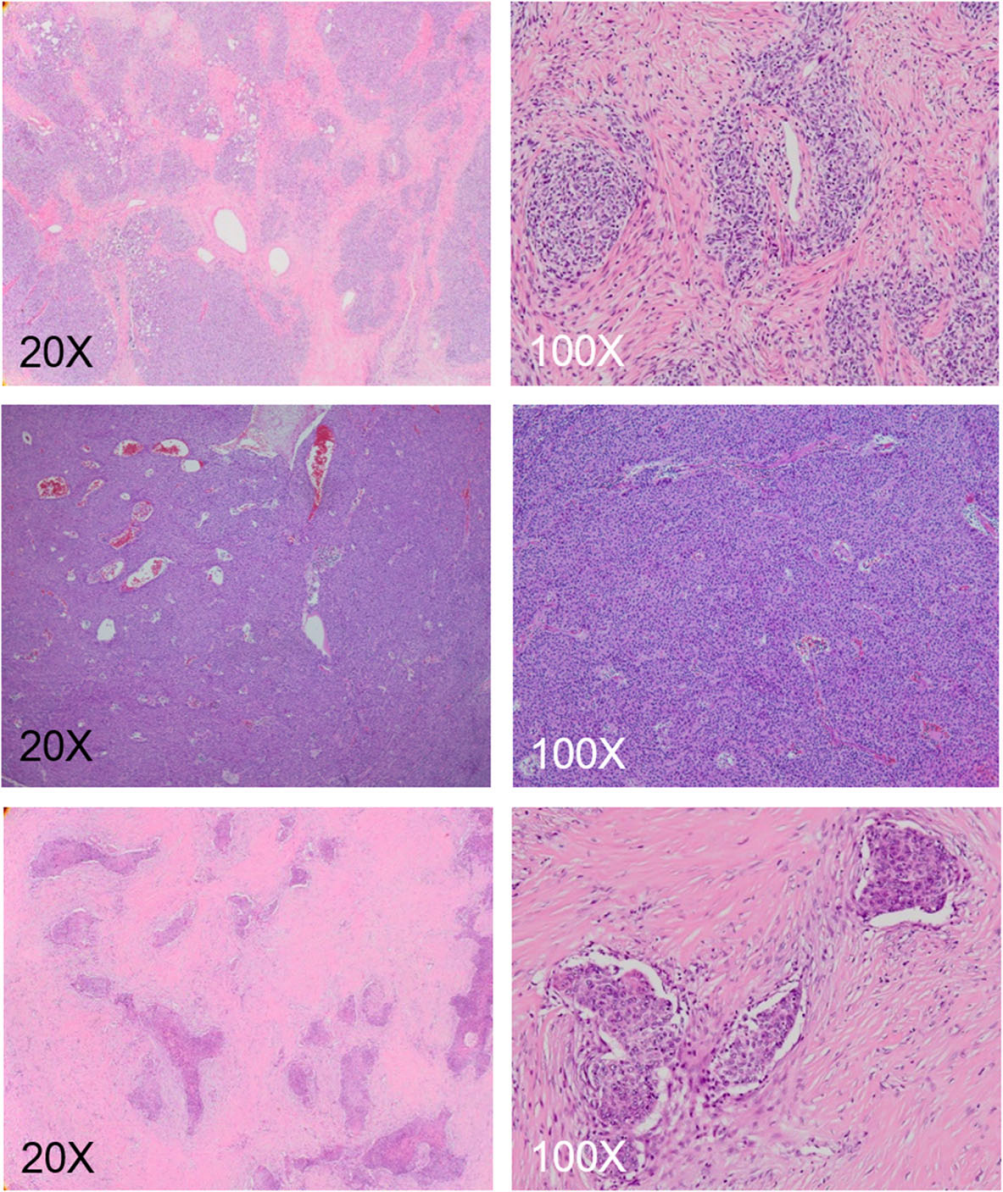
Stroma abundant group of TETs. Top row: Type A thymoma shows paucity of immature lymphocyte and abundant stromal component (20X & 100X). Middle row: Type B3 thymoma shows polygonal epithelial cells with minor component of lymphocytes (20X & 100X). Bottom row: thymic carcinoma shows angular nests of malignant epithelial cells embedded in a markedly increased and desmoplastic stroma (20X & 100X)

**Fig. 5:**
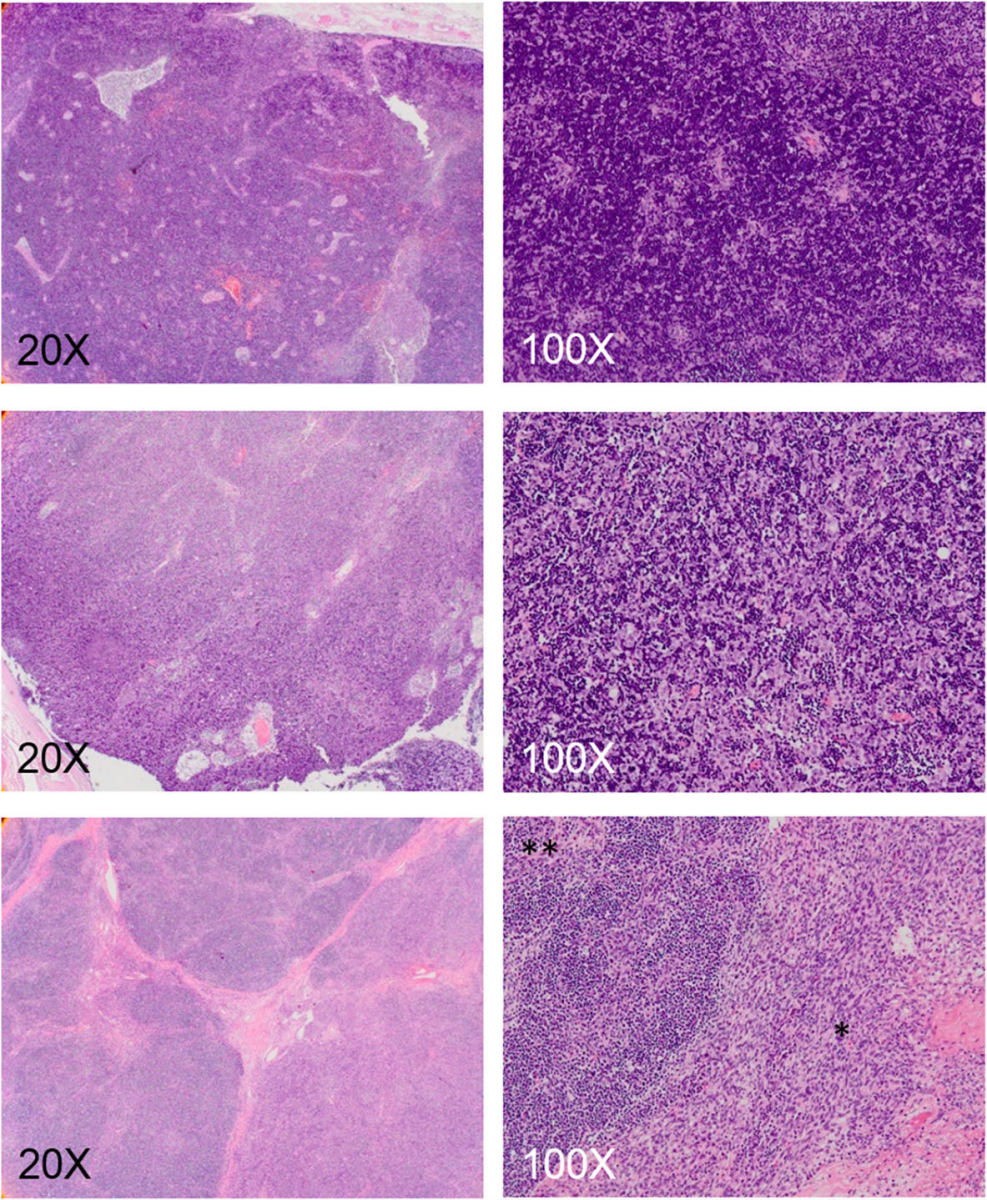
Stroma sparse group of TETs. Top row: Type B1 thymoma shows an abundance of immature lymphocytes (20X), and the lymphocytic population obscures the epithelial cells (100X). Middle row: Type B2 thymoma has a less dense lymphocytic infiltrate as comparison with type B1 thymoma (20X), and the epithelial cells are easily identifiable (100X). Bottom row: Type AB thymoma shows variable combination of lymphocyte-poor area (type A-like, *) and lymphocyte-rich area (type B-like, **) (20X & 100X)

**Fig. 6:**
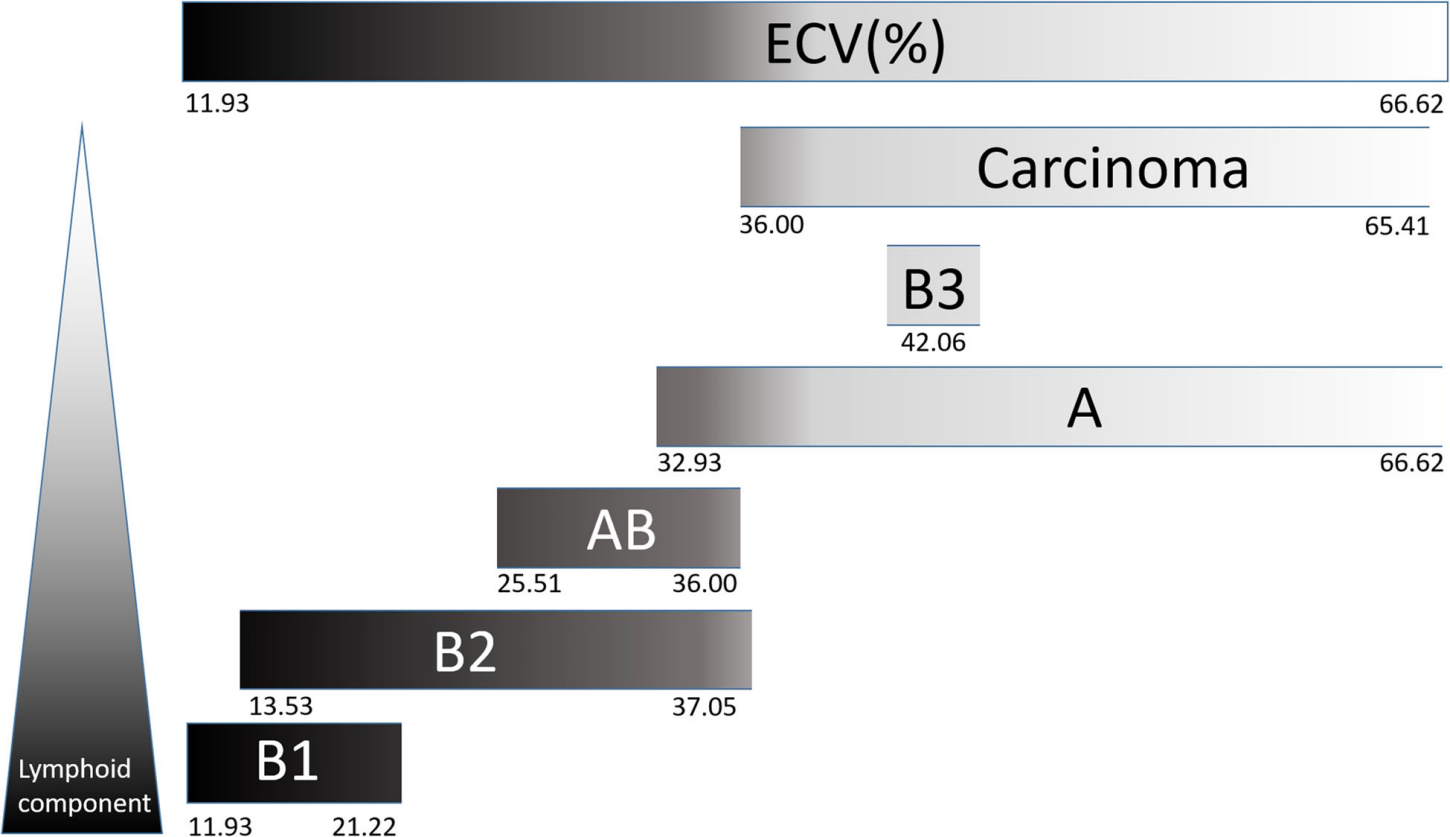
The ECV spectrum based on variable lymphoid component of different TETs. Type B1 thymoma shows the lowest ECV due to highest lymphocytic density. Type A thymoma, type B3 thymoma, and thymic carcinoma show high ECV due to sparse lymphoid density. And type AB and type B2 thymomas lie in the middle

T1 mapping with ECV measurement allows dichotomization of TETs into cellular and extra-cellular components, providing new frontiers for pathologic correlation. The use of ECV is preferable than native or postcontrast T1 as a biomarker, because ECV avoids confounders by taking into account the T1 behavior of blood, variable dosing and clearance of the contrast agent, and variation in the hematocrits. Besides, ECV is insensitive to systemic bias, the effects of renal function, anemia, or obesity [4–6]. Type A thymoma, type B3 thymoma, and thymic carcinoma showed higher ECV because of the sparse lymphocytes infiltrates (Fig. 4). On the other hand, type B1 and B2 thymomas belonged to the lymphocyte-rich thymomas, and type B1 thymoma showed the lowest ECV because of its dense lymphocystic population. Type AB thymoma has the components of type A and type B thymoma, and therefore the value depends on the proportion of either component (Fig. 5). The ECV values and lymphoid densities among different TETs are illustrated in Fig. 6. Notably, the type AB and type B2 thymoma show similar ECV value. It is difficult to differentiate thymic carcinoma from type A thymoma or type B3 thymoma based on ECV alone. Nonetheless, the histologic classification could be predicted with the radiographic invasiveness taken into consideration. For example, a radiographically invasive TET with high ECV would more likely be thymic carcinoma than type A thymoma, whereas TET with extremely low ECV would more likely be type B1 thymoma. Although our result demonstrates ECV has the ability to differentiate thymic carcinoma from high grade thymomas and low grade thymomas, however, from histopathological point of view, the change of ECV is not a linear correlation with histologic classification. In fact, the denotation and classification of thymic epithelial tumor have been a matter of heterogeneity more than a continuum of histologic spectrum. It has been increasingly recognized that thymomas are not as “pure” as previously assumed. For example, tumors denoted as type B2 thymoma might actually consist of small foci of type B1 thymoma, and thymic carcinoma could also contain type B3 thymoma [3]. According to our results, native T1 value or ECV could not differentiate histological subtypes of TETs just as pathologist could not differentiate TETs simply based on its lymphoid component under hematoxylin and eosin stain. The density of epithelioid cell, lymphoid cell and other immunohistochemistry were also mandatory to reach a precise diagnosis. Further investigation and development of MR imaging modality are mandatory to improve the diagnostic accuracy for thymic epithelial tumors. Combination of quantitative, qualitative data, variable imaging biomarkers, including T1 mapping, DWI, or dynamic contrast enhanced MR may help to predict cell type of thymic epithelial tumors or anterior mediastinal tumors. Taken together with clinical history, precise histologic diagnosis could be anticipated and unnecessary operation or biopsy could be avoided especially in patients with compromised performance status [21]. In patients undergoing surgery or chemoradiotherapy, follow-up for suspicious lesion using T1-mapping with ECV might help in depicting early disease relapse.

Our study had limitations. First, it was a single center retrospective study with small patient number and statistical power was therefore limited. Second, the image analysis was performed by manual ROI calculation of T1 value and ECV value, although excellent inter-observer reproducibility was confirmed in the measurement of the native T1 value and ECV. Third, our study did not compare predictive value of ECV with morphological features on routine MRI sequences. Fourth, because of the short follow-up period, oncologic outcome analysis between different histological types or lymphocyte abundance in TETs was not available. Future studies with larger number of patients are warranted to validate our results, and application of 3D volumetric calculation would be better for analysis if the tumor is heterogeneous.

## Conclusions

T1-mapping with ECV fraction measurement provides a non-invasive, reliable, and reproducible imaging tool for tissue characterization of TETs.

## Supplementary information

**Additional file 1: Table S1** MRI acquisition parameters. **Table S2** Patients’ treatment course and oncologic outcome.

## Data Availability

The datasets during and/or analysed during the current study available from the corresponding author on reasonable request.

## Abbreviations

ECV: Extracellular volume
TET: Thymic epithelial tumor
