# Supplementary material for: Extracellular volume fraction measurement correlates with lymphocyte abundance in thymic epithelial tumors
Source: Cancer Imaging. 2020 Oct 7;20:71. doi: 10.1186/s40644-020-00349-4 (PMC7539449; doi:10.1186/s40644-020-00349-4)
Supplement: Supplementary file 1 — Additional file 1: Table S1 MRI acquisition parameters. Table S2 Patients’ treatment course and oncologic outcome. [file 40644_2020_349_MOESM1_ESM.docx]

**Supplementary Table 1. MRI acquisition parameters**

| **Sequences** | **Scan plane** | **Voxel size (mm)** | **Slice thickness/ gap (mm)** | **TR/TE (ms)** | **FoV (mm)** | **Flip angle (^o^)** |
| --- | --- | --- | --- | --- | --- | --- |
| mDIXON (water, in-phase/ out-of-phase) | Transverse | 1.5 x 1.5 | 6/ -3 | 3.7/ 1.32/ 2.4 | 300 x 300 | 15 |
| T2W TSE FS | Sagittal | 1.2 x 1.7 | 8/ 1 | 800/ 71 | 350 x 328 | 90 |
| Cardiac-gated Double IR T2W | Transverse | 1.5 x 1.5 | 7/ 1 | 2 beats/ 73 | 250 x 250 | 90 |
| DWI (b= 0, 400, 800 sec/mm^2^) | Transverse | 3 x 3.02 | 7/ 1 | 2735/ 79 | 400 x 350 | 90 |
| MOLLI (pre-and post-CE) | Transverse | 3 x 1.3 | 7/ 1 | 2.9/ 1.34 | 250 x 250 | 35 |
| CE T1W FS | Transverse | 1.5 x 1.5 | 6/-3 | 3.6/ 1.32 | 300 x 300 | 10 |
| CE T1W FS | Sagittal | 1.3 x 1.3 | 6/-3 | 3.7/ 1.32 | 350 x 280 | 10 |

Abbreviations: TSE = turbo spin echo, FS = fat-suppressed, TE = echo time, TR = repetition time, IR = inversion recovery, DWI = diffusion-weighted imaging, MOLLI = modified Look-Locker inversion recovery, CE = contrast enhanced

**Supplementary Table 2. Patients’ treatment course and oncologic outcome**

| **Patients** | **Type** | **Masaoka stage** | **DFS (mo)** | **OS (mo)*** | **First-line treatment** | **Second-line treatment** | **Third-line treatment** | **Extrathymic malignancy** |
| --- | --- | --- | --- | --- | --- | --- | --- | --- |
| 1 | B1 | 4 |  | 26 | Sternotomy | CT | RT |  |
| 2 | B2 | 2 | 25 | 25 | VATS |  |  |  |
| 3 | AB | 2 | 25 | 25 | VATS |  |  |  |
| 4 | Carcinoma | 3 | 22 | 25 | Sternotomy | RT |  |  |
| 5 | AB | 1 | 25 | 25 | VATS |  |  |  |
| 7 | B2 | 2 | 25 | 25 | VATS | RT |  |  |
| 8 | Carcinoma | 4 |  | 25 | CT | RT |  |  |
| 6 | B2 | 1 | 24 | 24 | VATS |  |  |  |
| 9 | B2 | 2 | 24 | 24 | VATS | RT |  |  |
| 10 | B2 | 4 |  | 22 | CT | Sternotomy | CT |  |
| 26 | Carcinoma | 4 | 4 | 21 | CT | Sternotomy |  |  |
| 12 | Carcinoma | 3 | 16 | 21 | CT | Sternotomy | RT |  |
| 13 | Carcinoma | 3 | 21 | 21 | VATS | RT |  |  |
| 14 | B2 | 4 |  | 20 | Sternotomy | CT |  |  |
| 11 | Carcinoma | 3 | 19 | 19 | Sternotomy | RT |  |  |
| 15 | B2 | 3 | 19 | 19 | VATS | RT |  |  |
| 16 | AB | 1 | 18 | 18 | VATS |  |  |  |
| 17 | AB | 2 | 17 | 17 | Sternotomy |  |  |  |
| 18 | A | 1 | 17 | 17 | VATS |  |  |  |
| 19 | B2 | 4 |  | 17 | CT | Sternotomy | CT |  |
| 20 | A | 1 | 15 | 15 | VATS |  |  |  |
| 21 | B2 | 1 | 13 | 13 | VATS |  |  |  |
| 22 | B3 | 1 | 12 | 12 | Sternotomy |  |  |  |
| 23 | A | 2 | 11 | 11 | VATS |  |  | Endometrial cancer, stage I, underwent surgery in 2014, no relapse |
| 24 | Carcinoma | 4 |  | 11 | Sternotomy | CT |  |  |
| 25 | Carcinoma | 4 |  | 10 | CT |  |  |  |
| 27 | AB | 1 | 8 | 8 | VATS |  |  |  |
| 28 | AB | 1 | 8 | 8 | VATS |  |  |  |
| 29 | AB | 1 | 7 | 7 | VATS |  |  |  |
| 30 | B1 | 2 | 7 | 7 | VATS |  |  |  |
| 31 | A | 1 | 7 | 7 | Sternotomy |  |  |  |

Abbreviations: DFS = disease-free survival, OS = overall survival, mo = months, CT = chemotherapy, RT = radiotherapy, VATS = video assisted thoracoscopic surgery

*All patients survive until May, 2020.
